# Early and Accurate Prediction of Clinical Response to Methotrexate Treatment in Juvenile Idiopathic Arthritis Using Machine Learning

**DOI:** 10.3389/fphar.2019.01155

**Published:** 2019-10-07

**Authors:** Xiaolan Mo, Xiujuan Chen, Hongwei Li, Jiali Li, Fangling Zeng, Yilu Chen, Fan He, Song Zhang, Huixian Li, Liyan Pan, Ping Zeng, Ying Xie, Huiyi Li, Min Huang, Yanling He, Huiying Liang, Huasong Zeng

**Affiliations:** ^1^Department of Pharmacy, Guangzhou Women and Children’s Medical Center, Guangzhou Medical University, Guangzhou, China; ^2^Institute of Clinical Pharmacology, School of Pharmaceutical Sciences, Sun Yat-sen University, Guangzhou, China; ^3^Institute of Pediatrics, Guangzhou Women and Children’s Medical Center, Guangzhou Medical University, Guangzhou, China; ^4^Pediatric Allergy Immunology & Rheumatology Department, Guangzhou Women and Children’s Medical Center, Guangzhou Medical University, Guangzhou, China; ^5^Department of Medical, Guangzhou Women and Children’s Medical Center, Guangzhou Medical University, Guangzhou, China

**Keywords:** methotrexate, juvenile idiopathic arthritis, prediction model, machine learning, clinical response

## Abstract

**Background and Aims:** Accurately predicting the response to methotrexate (MTX) in juvenile idiopathic arthritis (JIA) patients before administration is the key point to improve the treatment outcome. However, no simple and reliable prediction model has been identified. Here, we aimed to develop and validate predictive models for the MTX response to JIA using machine learning based on electronic medical record (EMR) before and after administering MTX.

**Materials and Methods:** Data of 362 JIA patients with MTX mono-therapy were retrospectively collected from EMR between January 2008 and October 2018. DAS44/ESR-3 simplified standard was used to evaluate the MTX response. Extreme gradient boosting (XGBoost), support vector machine (SVM), random forest (RF), and logistic regression (LR) algorithms were applied to develop and validate models with 5-fold cross-validation on the randomly split training and test set. Data of 13 patients additionally collected were used for external validation.

**Results:** The XGBoost screened out the optimal 10 pre-administration features and 6 mix-variables. The XGBoost established the best model based on the 10 pre-administration variables. The performances were accuracy 91.78%, sensitivity 90.70%, specificity 93.33%, AUC 97.00%, respectively. Similarly, the XGBoost developed a better model based on the 6 mix-variables, whose performances were accuracy 94.52%, sensitivity 95.35%, specificity 93.33%, AUC 99.00%, respectively.

**Conclusion:** Based on common EMR data, we developed two MTX response predictive models with excellent performance in JIA using machine learning. These models can predict the MTX efficacy early and accurately, which provides powerful decision support for doctors to make or adjust therapeutic scheme before or after treatment.

## Introduction

Methotrexate (MTX) is the first line treatment for the majority of patients with juvenile idiopathic arthritis (JIA). However, the efficacy of MTX varies greatly among individuals, with about 30 to 70% of JIA patients being effective (Ruperto et al., 2004; Foell et al., 2010). Patients who respond to MTX poorly are given biologicals alone or in co-treatment with MTX. Biologics can lead to more efficient disease control, but abuse of biologics can result in high costs and serious adverse reactions. Additionally, it usually takes 3–6 months before a decision is made as to MTX efficacy (Martini et al., 2019). Patients receiving “trial-and-error” therapy for such a long time may delay treatment, resulting in irreversible joint damage and even adverse reactions. Therefore, early identification of whether the patient is effective before starting MTX and then selection of appropriate therapy (MTX alone or combined with biologics) are of great significance for preventing disease progression. This means that it is very necessary to establish an efficacy prediction model before the onset of MTX in JIA.

Although MTX has been used to treat JIA for a long time, being able to predict who will respond to MTX is still very limited. To date, only Bulatovic et al. (2012) reported a predictive model for MTX response to JIA. However, the limitations of this model are as follows: the prediction accuracy was not high (the area under the curve, AUC, was only 72%); model variables contained controversial single nucleotide polymorphisms (SNPs), which required additional and expensive testing, thus limiting its widely available in clinical application. Moreover, this study only employed one traditional logistic regression algorithm, which is not applicable to the modeling of non-independent variables. In addition to this study, other studies on MTX response to JIA were only limited to discovering which indicators would affect the efficacy of MTX. But they did not provide a model for clinical application, so that it could not be easily applied in clinical practice (Hinks et al., 2011; Yanagimachi et al., 2011; Cobb et al., 2014; Zajc Avramovic et al., 2017).

Therefore, a simple, efficient and accurate MTX response prediction model is urgently needed to provide references for clinicians before treatment. In recent years, the predictive model developed by machine learning based on electronic medical record (EMR) data has played an excellent role in disease diagnosis, treatment, and prognosis. For example, in our previous work, we used a machine learning technique to acquire pediatric EMR and developed an auxiliary decision-making system for diseases diagnosis, which is comparable to that of human physicians (Liang et al., 2019); Machine learning is also used to predict the efficacy and prognosis of diseases in other diseases (Motwani et al., 2017; Browning et al., 2019). Similarly, in rheumatoid diseases, researchers used machine learning to establish disease diagnosis classifier, mortality prediction model and MTX related hepatotoxicity automatic recognizer basing on EMR data (Liao et al., 2010; Lin et al., 2015; Lezcano-Valverde et al., 2017). These results provide practical tools for the management of patients. However, currently, there are no reports about the prediction model of MTX response in JIA using machine learning only basing on EMRs.

The purpose of this study is to develop simple, efficient and accurate models using machine learning for early predicting the efficacy of MTX in JIA based on integrating temporal features before and after starting MTX within three months.

## Methods

### Study Design and Population

We retrospectively collected the EMR data of children with JIA who visited Guangzhou Women and Children’s Medical Center from January 2008 to October 2018. Inclusion criteria were: (1) patients were new-onset and met the International League of Associations for Rheumatology criteria for JIA (Petty et al., 2004; Martini et al., 2019). (2) The onset age was 1–16 years old. (3) Patients received monotherapy with MTX for at least 3 months. (4) Co-treatment with non-steroidal anti-inflammatory drugs or corticosteroids were allowed. Exclusion criteria were: (1) combined therapy with other interfering drugs (e.g. biologic agents, sulfasalazine, etc.) within 3 months. (2) MTX therapy did not reach 3 months. (3) Serious missing of medical records. A total of 674 JIA children using MTX were screened out, but 362 patients were eventually included for modeling and validating. Furthermore, we continued to collect 13 JIA patients from November 2018 to January 2019 for external verification.

The study was performed according to the Helsinki declaration. Ethical approval was obtained from the ethics committee of this center (no. 2016021645). This study was a part of a large clinical trial (NCT81603203). All data were anonymous and no identifiable personal data of patients were available for the analysis. No additional informed consent was required.

### Assessment of MTX Clinical Response

Weekly MTX was given to all patients by either oral or subcutaneous route at 10–15 mg/m^2^. Baseline disease activity was calculated before MTX treatment. Early response to MTX was evaluated at 3 months after using MTX. Since it is a retrospective study, it is difficult to collect subjective features such as patient/parent and physician’s global assessment of disease. Therefore, JADAS or ACRpedi (see **Table 1** for the full name) scoring tools could not be applied to evaluate the response (Giannini et al., 1997; Consolaro et al., 2009). DAS44/ESR-3, a simplified standard related to the European League of Associations for Rheumatology criteria, was the most suitable choice for this retrospective study (Ranganath et al., 2007; Consolaro et al., 2009). The simplified formula of the disease activity is as follows: $y=0.53938\sqrt{RAI}+0.06465*SJC44+0.33\ln\left(ESR\right)+0.224$ (RAI, Ritchie articular index; SJC, Swollen joint count; ESR, erythrocyte sedimentation rate). The response was defined as a significant change of DAS44 scores from baseline to 3 months after starting MTX. Good response was defined as a significant decrease in DAS44 (>0.6), while a decrease of ≤0.6 was non-response.

**Table 1:** The full name and abbreviation name of variables.

Full name of variables	Abbreviation name	Full name of variables	Abbreviation name
Age of methotrexate start	Age of MTX start	Hemoglobin	HGB
Age onset	Age onset	Indirect bilirubin	IBIL
Albumin	ALB	Immune globulin A	IgA
Alanine transaminase	ALT	Immune globulin E	IgE
Anti-cyclic citrullinated peptide	Anti-CCP	Immune globulin G	IgG
Active partial thrombin time	APTT	Immune globulin M	IgM
Aspartate aminotransferase	AST	JIA subtype	JIA subtype
Complement 3	C3	Lymphocyte	LYM
Complement 4	C4	Neutrophil	NEUT
CD16+CD56+	CD16+CD56+	Platelet	PLT
CD19+	CD19+	Prothrombin time	PT
CD3+Abs	CD3+Abs	Red blood cell	RBC
CD3+CD4+	CD3+CD4+	Rheumatoid factor-IgG	RF-IgG
CD3+CD8+	CD3+CD8+	Serum creatinine	SCr
C-reactive protein	CRP	Swollen joint count	SJC
Direct bilirubin	DBIL	Total bilirubin	TBIL
The first dose of MTX on the start	Dose0	Helper T cells/Suppressor T cells	Th/Ts
Erythrocyte sedimentation rate	ESR	Time interval	Time interval
Ferritin	FER	Tender joint count	TJC
Fibrinogen	FIB	Thrombin time	TT
Gender	Gender	Urea	Urea
Blood glucose	GLU	White blood cell	WBC
Hematocrit	HCT	Weight	Weight
Ritchie articular index	RAI	C-reactive protein near 3 months after administration	CRP/3m

CD, Cluster of differentiation cell; CD3+Abs, the absolute value of T cell with Cluster of differentiation 3; CD3+CD4+, the ratio of CD4+ divided by CD3+.

Variables with the suffix “/3m” are those collected within 3 months after administration of MTX. For example, CRP/3m refers to the CRP variable collected within 3 months after administration.

### Clinical Variables

**Figure 1:**
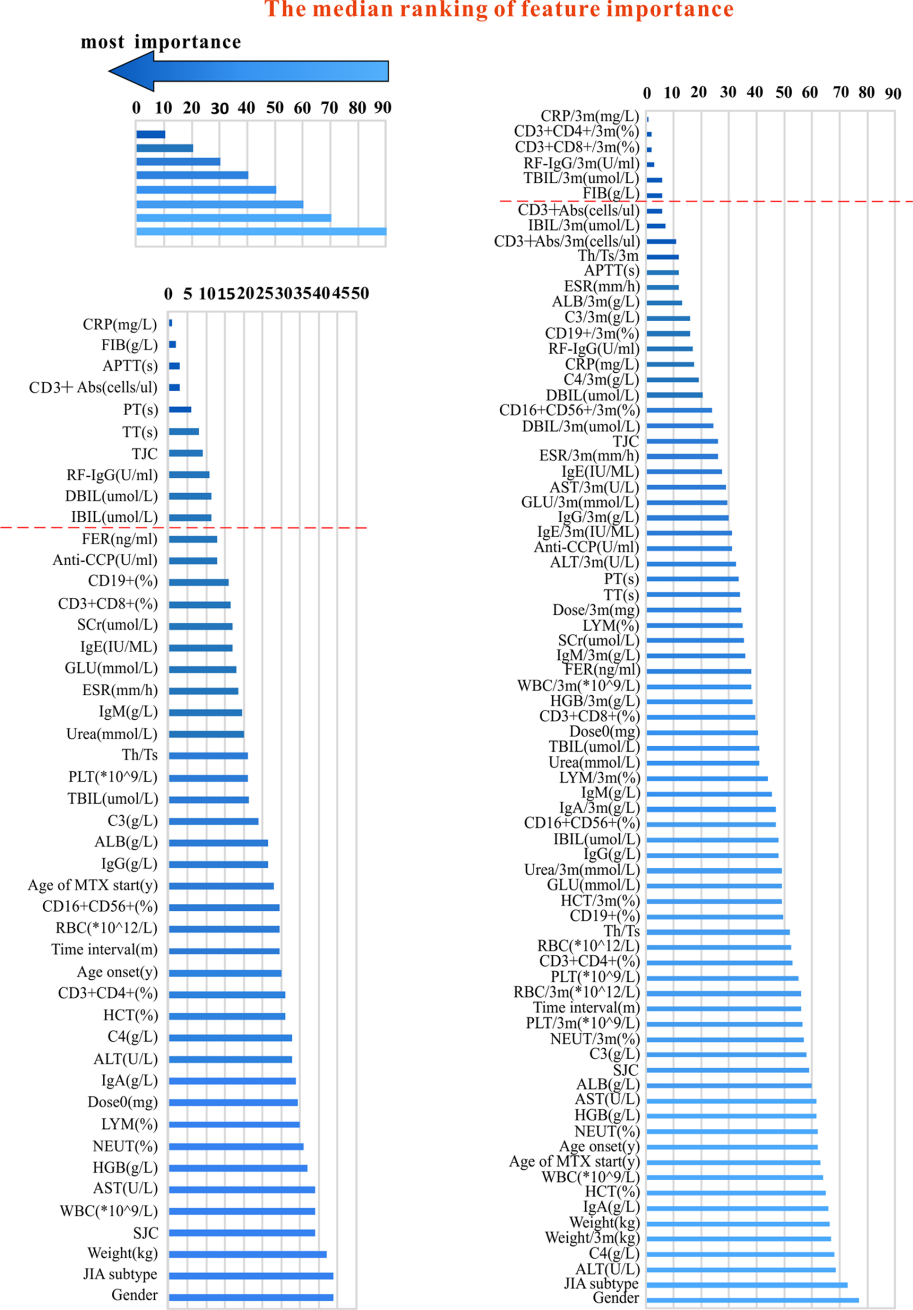
The variables used for modeling and their importance ranking (in order of median importance). The left part of the figure shows the variables used in the pre-administration variables model. The right part of the figure shows a mixture of variables before and after administration (variables collected within 3 months after administration with MTX). The shorter the transverse column (i.e. the smaller the value), the greater importance of the median ranking of the variable (see the top and left part).

All data were collected from EMRs before administration of MTX (baseline) and within 3 months after administration (nearly 3 months). We collected a lot of variables, including: joint conditions [tender joint count (TJC), SJC, RAI, and JIA subtypes (oligoarticular, polyarticular, and other subtypes), joint imaging, etc.], the acute phase of inflammatory products [C-reactive protein (CRP), ESR], demographic data (age, gender, etc.), immune-related indicators [rheumatoid factor (RF), rheumatoid factor IgG (RF-IgG), antinuclear antibodies, anti-cyclic citrullinated peptide antibody, etc.], kidney function, liver function [total bilirubin (TBIL), direct bilirubin (DBIL), indirect bilirubin (IBIL), etc.], blood coagulation function [active partial thrombin time (APTT), prothrombin time (PT), thrombin time (TT), fibrinogen (FIB), etc.], blood routine testing, relevant lymphocytes (CD3+abs, CD3+CD4+, CD3+CD8+, etc.) and other data. See **Table 1** for a list of all variables. Because some variables were seriously missing, they were not be used for modeling. All variables used for modeling are shown in **Figure 1**.

### Machine Learning

**Figure 2:**
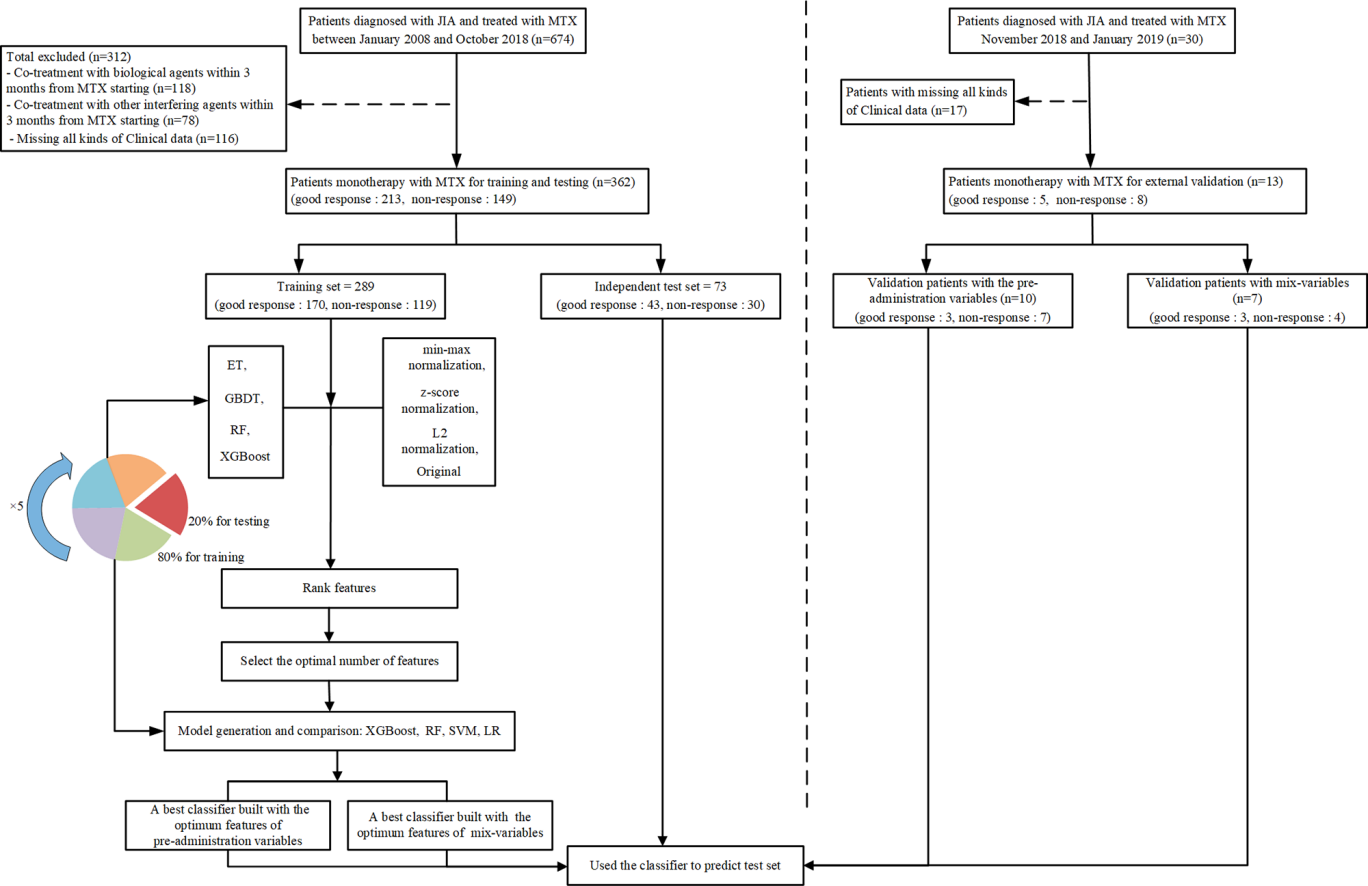
The flowchart of model developing and validation. ET, extremely randomized trees; GBDT, gradient boosting decision tree; RF, random forest; XGBoost, extreme gradient boosting; SVM, support vector machine; LR, logistic regression.

We collected two different sets of variables. Thus, two groups of models were established based on variables before the onset of MTX and mix-variables within 3 months after starting MTX respectively, for finding the best model. In the first group of models, referred to as pre-administration variables models (MTX-A), we included 46 variables (see the left part of **Figure 1**). In the second group of models, referred to as mix-variables models (MTX-B), we extended MTX-A by adding 32 new variables (see the right part of **Figure 1**). The main process can be divided into three steps: (1) data processing, (2) feature selection, (3) model generation and validation. Five-fold stratified cross-validation was used for assessing the performance and general error estimation of feature selection and model generation. **Figure 2** shows the flowchart of this work. Machine learning techniques were implemented in Python 3 (Python 3.6.5) using the package Scikit-learn (Scikit-learn 0.19.1).

### Data Preprocessing

Some variables have been removed with >30% missing rates. In order to get a higher quality of the dataset, the missing values were filled with mean values of a group stratified by MTX response. For example, we used mean values of good response and non-response group to respectively fill the data of CRP in different outcome groups.

### Feature Selection

Appropriate feature subsets were selected using ensemble models, including extremely randomized trees (ET), gradient boosting decision tree (GBDT), random forest (RF) and extreme gradient boosting (XGBoost). Firstly, data transformation was carried out on continuous variables to form four kinds of data: min-max normalization, z-score normalization, L2 normalization and original. Secondly, the above four algorithms were used to analyze the four forms of data and build 16 models using five-fold stratified cross-validation, so as to obtain the median importance ranking of variables in all models which is as the final features importance ranking. Finally, the next aim is to determine the feature set with the least variables but the highest predictive accuracy. The XGBoost algorithm was used to find the minimum-size list of features by forwarding feature selection, as follows: (1) beginning with the head of the ranked list of variables (the most important variable), XGBoost algorithm iteratively generates a new model by adding one variable at a time, and calculates its classification accuracy. (2) The list with the minimum size and optimum accuracy is therefore selected.

### Model Generation and Validation

A cohort of 362 patients was randomly split into the training set and test set according to the ratio of 80:20. XGBoost, RF, support vector machine (SVM) and logistic regression (LR) algorithms were applied to develop classifiers respectively in our study. The classifiers were trained on the training set (n = 289), using the training set feature values (the minimum-size list of features) as input. Thus, each set of variables had 4 types of classifiers. Five-fold stratified cross-validation was used for internal validation. After training, classifiers were asked to predict the response of the test set (n = 73). For each set of variables, the four classifiers were compared with each other in terms of accuracy, and then the best classifier was selected as the final predictor. We further performed an external validation of the above classifiers with the subsequent collection of 13 patients.

## Results

### Patient Characteristics

A total cohort of 362 patients with JIA was included in developing models, and 13 patients were subsequently collected to external validate the best model. **Table 2** describes the baseline characteristics of our study population. According to DAS44/ESR-3 simplified standard, 213 patients were rated as good response and 149 patients as non-response.

**Table 2:** Baseline patient characteristics.

Characteristics	Data (n = 362)
Gender, n (male/female)	211/151
Age of MTX start, years, (mean ± SD)	6.7 ± 3.4
Age of disease onset, years, (mean ± SD)	6.3 ± 3.4
Time interval*, months, (mean ± SD)	5.6 ± 2.7
Polyarticular JIA, n	101
Oligoarticular JIA, n	186
Other types of JIA, n	75
Tender joint count, median (range)	3(0–36)
Swollen joint count, median (range)	4(0–36)
ESR, mm/h, (mean ± SD)	36.22 ± 33.34
CRP, mg/L, (mean ± SD)	24.81 ± 33.32
RF-IgG, U/ml, (mean ± SD)	23.68 ± 52.55
MTX dose at start, mg/m^2^/wk, median (range)	5.0(0.5–18.0)

*Time interval, the time from disease onset to initiation of MTX treatment.

### Feature Selection

**Figure 3:**
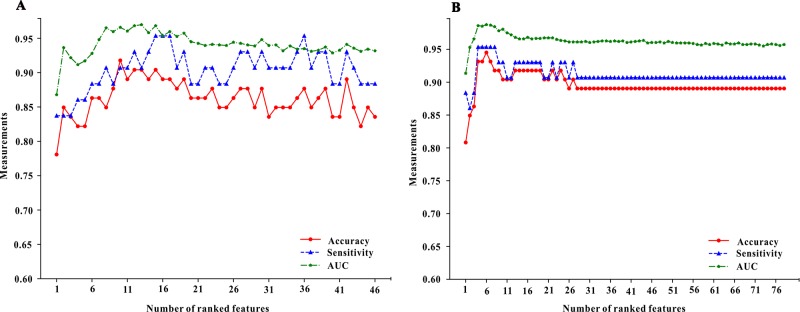
The forward feature selection results for the model of pre-administration variables (the **A** part of figure 3) and the model of mix-variables before and after administration (the **B** part of figure 3). We examined the predictive performance of the most prominent feature and identified the point at which there was no considerable gain in accuracy, sensitivity, and area under the curve (AUC), when adding the feature of the next highest ranking one to the model. The optimum values were obtained when these three measurements defined the most discriminative features.

Median importance ranking of all variables before using MTX and mix-variables before and after administering with MTX were shown in **Figure 1** (left part and right part). The XGBoost algorithm was applied for selecting the minimum size and optimum accuracy features subset, and the results of this process were shown in **Figure 3**. We examined the predictive performance of the most prominent feature and identified the point at which there was no considerable gain in accuracy, sensitivity, and AUC, when adding the feature of the next highest ranking one to the model. The optimum values were obtained when these three measurements defined the most discriminative features. In the MTX-A predictors, the three measurements reached the optimum when 10 feature subsets were selected (see **Figure 3A**). The 10 selected significant variables are listed above the dotted red line in the left part of **Figure 1**. In the MTX-B predictors, the three measurements achieve maximum performance when 6 feature subsets were screened out (see **Figure 3B**). Variables above the dotted red line in the right part of **Figure 1** are these 6 features. The degree of contribution of all the above selected variables to response and the formulas behind modeling were described in detail in the **Supplementary**.

### Model Performance and Comparison

**Figure 4:**
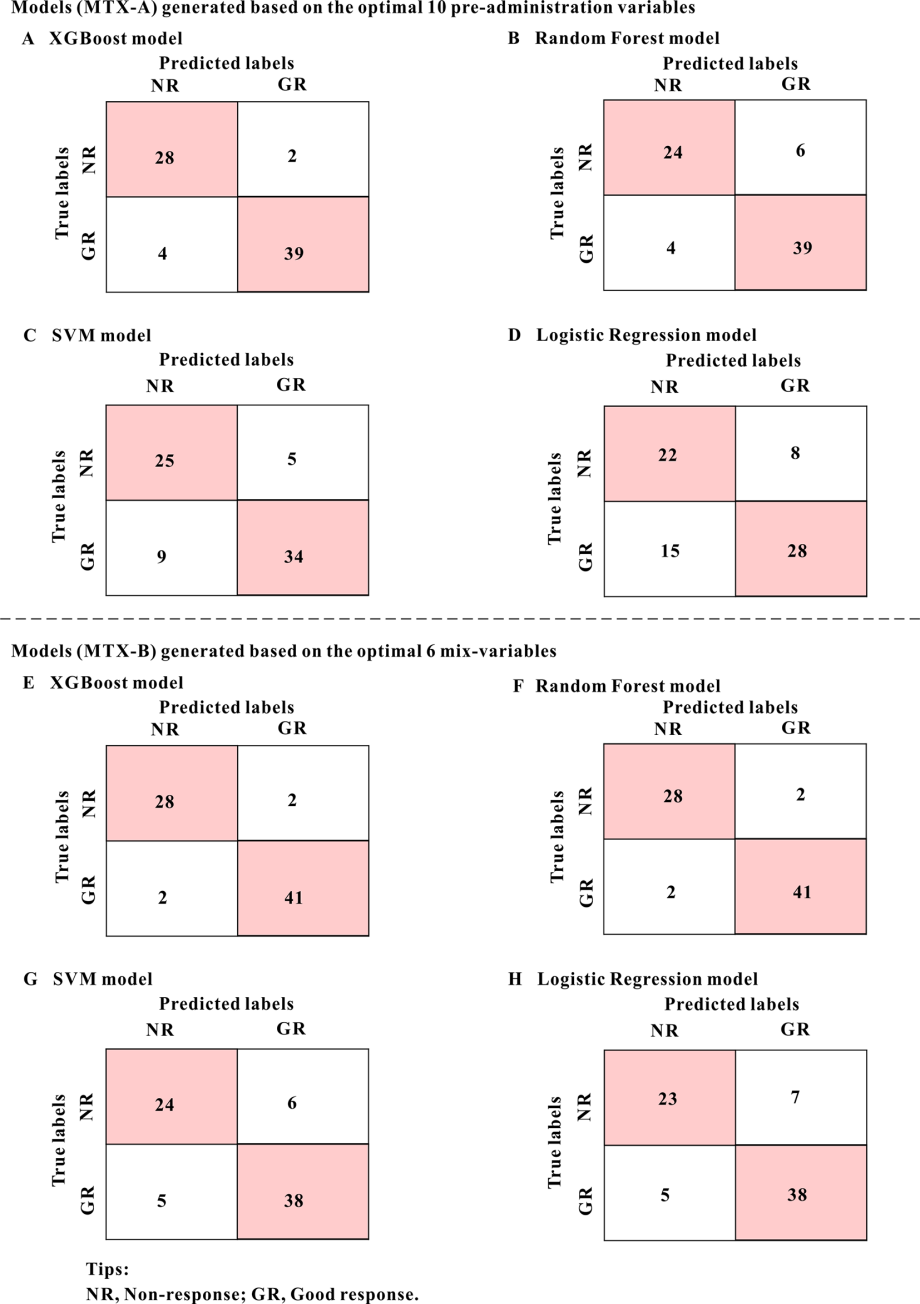
The mixed matrix results of each model in the MTX-A and MTX-B predictors of the test set. For example, when the true lables were NR(non-response) and predicted lables were NR, it indicated that the number of NR was correctly predicted. However, when true lables were NR, and predicted labels were GR (good response), it indicated the number of NR incorrectly predicted to GR. As can be seen from the figure, the predicted values of model A (XGBoost model) and model E (XGBoost model) are the closest to the real values, indicating the best prediction performance. The numbers in the pink grids represent the number of cases that were accurately predicted.

**Table 3** shows the classification accuracy results of the models which were evaluated using the test set. Of the MTX-A and MTX-B predictors, both the XGBoost models showed the best predictive performances. Therefore, the XGBoost models were selected as the final predictors. The performance of MTX-A XGBoost predictor was as follows: sensitivity 90.70% (95%CI: 82.0–99.4%), specificity 93.33% (95%CI: 84.4–100%), accuracy 91.78% (95%CI: 85.5–98.1%) and AUC 0.97. However, the MTX-B XGBoost predictor have a better performance, which achieves sensitivity 95.35% (95%CI: 89.0–100%), specificity 93.33% (95%CI: 84.4–100%), accuracy 94.35% (95%CI: 89.3–99.7%) and AUC 0.99. **Figure 4** shows the mixed matrix results of each model in the MTX-A and MTX-B predictors of the test set.

**Table 3:** The classification performance results of the models.

Data set	Model	Sensitivity (%)	Specificity (%)	Accuracy (%)	PPV (%)	NPV (%)	AUC
MTX-A	XGBoost	90.70	93.33	91.78	95.12	87.50	0.97
	RF	90.70	80.00	86.30	86.67	85.71	0.95
	SVM	79.07	83.33	80.82	87.18	73.53	0.87
	LR	65.12	73.33	68.49	77.78	59.46	0.80
MTX-B	XGBoost	95.35	93.33	94.52	95.35	93.33	0.99
	RF	95.35	93.33	94.52	95.35	93.33	0.98
	SVM	88.37	80.00	84.93	86.36	82.76	0.81
	LR	88.37	76.67	83.56	84.44	82.14	0.83

MTX-A, pre-administration variables prediction models; MTX-B, mix-variables models with features collected before and after administered with MTX within 3 months; PPV, positive predictive value; NPV, negative predictive value; AUC, area under the curve; XGBoost, extreme gradient boosting; RF, random forest; SVM, support vector machine; LR, logistic regression.

### External Verification and Clinical Application

Data of 13 patients newly collected were applied to externally validate the two XGBoost predictors. The performances of MTX-A XGBoost predictor were sensitivity 100.0%, specificity 86.1% and accuracy 90.0%, respectively. But the MTX-B XGBoost predictor got better performance results, with 100% sensitivity, specificity, and accuracy. Furthermore, we applied these predictors to two randomly selected patients to predict their outcomes. We input their clinical variables into the two predictors. Both models produced correct predictive outcomes (see **Table 4**).

**Table 4:** The application of XGBoost predictors for clinical patients to predict their response.

MTX-A Predictor	Patient name	Input variables														Output
		CRP (mg/L)	FIB (g/L)		APTT (s)		CD3+Abs (cells/ul)	PT (s)	TT (s)	TJC	RF-IgG (U/ml)	DBIL (umol/L)		IBIL (umol/L)		
	AAA	35.30	2.77		34.20		2,454.46	12.00	10.90	1	21.50	1.30		4.80		Non- response
	BBB	150.00	4.71		26.22		3,309.00	14.40	10.90	2	5.90	2.60		2.70		Good response
MTX-B Predictor	Patient name	Input variables														Output
		CRP/3m (mg/L)		CD3+CD4+/3m(%)		CD3+CD8+/3m (%)			RF-IgG/3m(U/ml)		TBIL/3m (umol/L)		FIB(g/L)			
	AAA	49.60		38.00		50.00			11.70		6.17		2.77			Non- response
	BBB	32.30		34.44		26.77			3.90		7.60		4.71			Good response

In this table, variables were from clinical determination. The patient named AAA was responded to MTX in clinic practice, but the patient named BBB was not responded to MTX. We input their clinical variables into the two predictors. Both models produced correct predictive outcomes. Output means the prediction results of the MTX response.

## Discussion

We developed and validated two prediction models for MTX response in a large JIA cohort according to EMR data using machine learning. Models developed by XGBoost showed the best performance. CRP, CD3+Abs, RF-IgG, TJC, DBIL, IBIL, APTT, PT, TT, and FIB were variables screened out by the pre-administration variables model; and the mix-variables model filtered out features as follows: CRP/3m, CD3+CD4+/3m, CD3+CD8+/3m, RF-IgG/3m, TBIL/3m, and FIB, which were collected before and after administering MTX. The pre-administration variables model could accurately identify ninety-seven percent of patients whether responded to MTX (AUC 97%); 99% of patients were distinguished by the mix-variables model (AUC 99%).

To our knowledge, this is the first article to establish an accurate and easy-to-use predictive model for the efficacy of MTX in JIA based solely on EMRs using machine learning. Bulatovic et al. (2012) reported the first and so far the only one efficacy prediction model, which including variables of SNPs and ESR. The identified accuracy, sensitivity, specificity, positive predictive value, negative predictive value were 72, 78, 49, 83, and 41%, respectively. These results were all lower than ours. SNPs are important in precision medicine. However, a GWAS study found no direct correlation between SNPs related to the pathways of MTX and the efficacy in 759 JIA patients (Cobb et al., 2014). This suggests that SNPs may not necessary in revealing the outcome of JIA (Albers et al., 2009; Roszkiewicz and Smolewska, 2017). Moreover, the expression and activity of enzymes in children may be affected by growth, and the genotype may not directly reflect the phenotype (Zhao et al., 2010; Roszkiewicz and Smolewska, 2017). In addition, genotype detection increases the cost and time, which is not as convenient, efficient and cheap as conventional detection. These suggest that clinical phenotypic variables may be more important in influencing outcomes (Roszkiewicz and Smolewska, 2017). Our results verified the above view.

Of course, the low prediction accuracy of Bulatovic’s study may be because of using only the traditional logistic regression, which may not be the optimal method. Machine learning is hot in recent years, which has gained remarkable achievements in biomedicine (Austin et al., 2013; Lin et al., 2015; Motwani et al., 2017; Browning et al., 2019; Liang et al., 2019). Specifically, machine-learning approaches may offer advantages over conventional techniques. The advantages of machine learning over traditional modeling methods are as follows: (1) machine learning can deal with more complex, high-dimensional and interactive variables, but the latter has limited ability to fix those problems. (2) Traditional modeling has poor generalization, though the former can model with strong generalization and better accuracy (Kruppa et al., 2012; Lee et al., 2018). Therefore, several advanced machine learning methods including SVM, RF, and XGBoost as well as LR were applied to model and compare the results in our study. Of our results, the performances of XGBoost models were the best, and the results of LR models were mostly poor. Further, the performances of those four modeling methods were all better than those of Bulatovic’s study. These results also confirmed that XGBoost could effectively avoid overfitting and improve prediction performance. LR, as a kind of traditional analysis, may appear low-fitting (Lee et al., 2006). Therefore, when dealing with similar classification problems, it may be more appropriate to select advanced algorithms.

In addition to the above Bulatovic’s study, other literature also explored which variables were associated with the efficacy of MTX (Yanagimachi et al., 2011; Cobb et al., 2014; Zajc Avramovic et al., 2017). But they did not establish a model, which could not be applied easily in clinical. For instance, some studies showed SNPs were associated with MTX efficacy (Hinks et al., 2011; de Rotte et al., 2012; Zajc Avramovic et al., 2017) clinical variables like TJC, ESR, CRP, etc. were also correlated with MTX response (Albers et al., 2009; Yanagimachi et al., 2011; Cobb et al., 2014; Franova et al., 2016). Variables considered in our study (n = 78) were more than and mostly different from those of the reported studies (Albers et al., 2009; Yanagimachi et al., 2011; Cobb et al., 2014; Franova et al., 2016). TJC, CRP and RF-IgG screened out by our study were reported to associate with disease activity, prognosis, efficacy of rheumatoid arthritis or JIA (Zborovskii et al., 1999; Cabral et al., 2005; Jaskowski et al., 2010). CD3+, CD4+, and CD8+ are the most important T lymphocytes, which are widely distributed in the joint synovial membrane and fluid and play an important role in the pathogenesis and classification of JIA (Finnegan et al., 2011; Hui et al., 2012; Van Nieuwenhove et al., 2019). One mechanism of MTX action is to regulate or inhibit T cell immunity to achieve the therapeutic effect (Johnston et al., 2005). In this study, these cells were contributed to MTX outcome, which is consistent with the MTX effect mechanism and similar to some reports (Isaacs, 2007; Bulatovic Calasan et al., 2015). Liver function variables (TBIL, DBIL, and IBIL) were also screened out. It is well-known that MTX is metabolized by the liver, so its pharmacokinetics will be affected by liver function, which will then affect pharmacodynamics. Coagulation markers (APTT, PT, TT, and FIB) also contributed to MTX efficacy in our study. We know ESR is an important variable in calculating disease activity and MTX efficacy. While ESR depends mainly on plasma FIB (Bedell and Bush, 1985). Additionally, FIB affects the adhesion, spread, proliferation of endothelial cells, and the repair of joint synovial tissue. So FIB may have an indirect contribution to MTX treatment (Carney et al., 1992; Cid et al., 1993; Grober et al., 1993). As for the degree of contribution of all the above selected variables to the outcome (efficacy), we described details in the **Supplementary**. It can be seen from the **Supplementary** that CRP was the most significant variable for the pre-administration model, while for the mix-variables model, RF-IgG/3m was the most important.

Additionally, from our results, the mix-variables model was better than the pre-administration variables model. This indicated that temporal features after administration but before evaluating efficacy also had an important influence on the treatment outcome. Just as variables before and during pregnancy can both have an impact on pregnancy outcome. This suggests that in addition to considering the influence of pre-administration variables, the efficacy should be evaluated in combination with post-administration features. The limits of this study were the relatively small sample size, insufficient representativeness of externally verified samples and retrospective research. The next step is to conduct prospective studies to model and validate.

## Conclusions

In summary, for the first time, based on EMR we used advanced machine learning to establish two early predictive models for the MTX efficacy in JIA, including pre-administration variables model and mix-variables model. The latter model performed better. The variables screened by the models were closely related to the pathogenesis of diseases, pharmacokinetics and pharmacodynamics of MTX, and could be fully explained. Interestingly, the coagulation indicators filtered out by our models may indicate the new pathogenesis of JIA and the unelucidated mechanism of MTX. This model is simple, efficient and accurate, and can be easily generalized by clinicians and pharmacists to make early treatment decisions to patients of different ethnic groups.

## Data Availability Statement

The datasets generated for this study are available on request to the Corresponding Author.

## Ethics Statement

The studies involving human participants were reviewed and approved by drug clinical research ethics committee of Guangzhou women and children’s medical center. Written informed consent to participate in this study was provided by the participants’ legal guardian/next of kin. Written informed consent was Obtained From the Minor(S)’ Legal Guardian/Next of kin for the publication of any potentially identifiable images or data included in this article.

## Author Contributions

Conceptualization: XM, YH, HL (16th author) and HZ. Data curation: XM, HWL, YC, FH, SZ and HL (13th author). Formal analysis: XC, HXL, LP, HL (16th author) and HZ. Funding acquisition: XM and MH. Investigation: XM, HWL, YC and FH. Methodology: XM, XC, JL, HXL and LP. Project administration: XM, MH, YH, HL (16th author) and HZ. Resources: HWL, SZ, PZ, YX and HZ. Software: XC and HXL. Supervision: XM. Validation: PZ and YX. Writing — original draft: XM and XC. Writing — review and editing: XM, XC, HWL, JL, FZ, YH, HYL and HZ.

## Funding

This research was supported by grants from the National Natural Science Foundation of China (grant no. 81603203), the National Key Research and Development Program (grant no. 2017YFC0909303), the National Key Research and Development Program (Grant No. 2016YFC0905001), Health Commission of Guangdong Province (Grant No. A2016400), Guangdong Pharmaceutical Association Program (Grant No. 2015FS10 and 2015SW05), Guangzhou Institute of Pediatrics/Guangzhou Women and Children’s Medical Center (Grant No. YIP-2018-020).

## Conflict of Interest

The authors declare that the research was conducted in the absence of any commercial or financial relationships that could be construed as a potential conflict of interest.
