# Supplementary material for: Early and Accurate Prediction of Clinical Response to Methotrexate Treatment in Juvenile Idiopathic Arthritis Using Machine Learning
Source: Front Pharmacol. 2019 Oct 7;10:1155. doi: 10.3389/fphar.2019.01155 (PMC6791251; doi:10.3389/fphar.2019.01155)
Supplement: Supplementary file 1 [file Table_1.docx]

**Supplementary**

**Details of the formulas behind predictive models and the contribution of variables to the outcome (efficacy)**

The model constructed using logistic regression (LR) method can be formulated. Thus, the LR model can reflect the direction and magnitude of the effect of the variables in the two predictive models on the outcome. The models built using support vector machines (SVM), random forest (RF) and extreme gradient boosting (XGBoost) are black-box, which can not reflect the directional effect of the variables included in the models on outcome clearly. SVM (Devos *et al.*, 2009, Cortez *et al.*, 2013) although it combines nearest neighbor learning and linear regression, the multidimensional data involved in this study is linearly inseparable. The data is mapped to a high-dimensional space using a radial based function (RBF), and then a hyperplane is set up in the space for further classification. The mapping is a black box, so it is impossible to draw a specific formula. Additionally, the RF (Biau *et al.*, 2016) and the XGBoost (Babajide *et al.*, 2016) are also the black-box algorithms, which means that a simple formula cannot be drawn.

The LR formula was as follows:

$$P_{outcome=1(good response)}=\frac{1}{1+e^{-TX}} (1)$$

$$\mathrm{TX}_{(MTX-A)}=-0.01-\left( 6.39e-03 \right)\times CRP+\left( 3.63e-01 \right)\times FIB-\left( 7.61e-02 \right)\times APTT+\left( 2.53e-04 \right)\times\left( Cd3+\mathrm{Abs} \right)-\left( 6.52e-02 \right)\times PT+\left( 6.83e-02 \right)\times TT+\left( 1.60e-01 \right)\times TJC-\left( 8.76e-03 \right)\times\left( \mathrm{RF}-\mathrm{IgG} \right)-\left( 2.04e-01 \right)\times DBIL+\left( 1.66e-01 \right)\times IBIL (2)$$

$$\mathrm{TX}_{(MTX-B)}=1.12-0.14\times(CRP/3m)-0.07\times(CD3+CD4+/3m)-0.01\times(CD3+CD8+/3m)+(0.03e-01)\times(RF-IgG/3m)+0.16\times(TBIL/3m)+0.53\times FIB (3)$$

As shown in formula (1), this is the mathematical expression of LR method (***P*** represents the probability of the prediction results that MTX efficacy is a good response, TX is the dot product of the variable and the corresponding regression coefficient). Thus, from the formula (1) and (2), the LR model of pre-administration variables models (MTX-A) can be obtained. Similarly, the formula (1) combined with the formula (3), is the LR model of mix-variables models (MTX-B). As for LR algorithm (Stoltzfus, 2011), the positive regression coefficient indicates that the corresponding variable is positively correlated with *P*. The negative regression coefficient indicates that the corresponding variable is negatively correlated with *P*. The larger the regression coefficient is, the greater the influence of the corresponding variable on the *P* result will be. In the LR model of MTX-A predictor, FIB, Cd3+Abs, TT, TJC and IBIL were positively correlated with *P*, while CRP, APTT, PT, RF-IgG and DBIL were negatively correlated with *P*. Among them, FIB and DBIL had a greater influence on *P* results. In the LR model of MTX-B predictor, RF-IgG/3m, TBIL/3m and FIB were positively correlated with *P*, while CRP/3m, CD3+CD4+/3m and CD3+CD8+/3m were negatively correlated with *P*, and FIB, TBIL/3m and CRP have a greater impact on *P* results.

Table 1 shows the important contribution degree of variables in models of MTX-A and MTX-B predictor based on RF and XGBoost algorithm (A larger value indicates that the greater the contribution of the variable to the model results, the more important it is, but the direction of influence cannot be judged.). In the four models of table 1, the contribution of CRP and CRP/3m to the prediction results of the model were relatively large, even the largest. In addition, the RF-IgG/3m variable contributed the most in the XGBoost model of MTX-B predictor. Therefore, in the XGBoost model of MTX-A and MTX-B predictor, CRP, CRP/3m and RF-IgG/3m are most important for the prediction of the MTX efficacy.

**Table 1. The important contribution degree of variables in both** **MTX-A and MTX-B predictor**

| **MTX-A**  **Predictor** | **Method** | **Important contribution degree of variable** | | | | | | | | | | | | |
| --- | --- | --- | --- | --- | --- | --- | --- | --- | --- | --- | --- | --- | --- | --- |
|  |  | CRP  (mg/L) | FIB  (g/L) | | APTT  (s) | | CD3+Abs(cells/ul) | PT  (s) | TT  (s) | TJC | RF-  IgG  (U/ml) | DBIL  (umol/L) | | IBIL  (umol/L) |
|  | XGBoost | 0.19 | 0.09 | | 0.13 | | 0.13 | 0.05 | 0.06 | 0.07 | 0.10 | 0.09 | | 0.08 |
|  | RF | 0.18 | 0.13 | | 0.16 | | 0.14 | 0.07 | 0.05 | 0.05 | 0.08 | 0.07 | | 0.06 |
| **MTX-B**  **Predictor** | **Method** | **Important contribution degree of variable** | | | | | | | | | | | | |
|  |  | CRP/3m  (mg/L) | | CD3+CD4+/3m(%) | | CD3+CD8+/3m  (%) | | | RF-IgG/3m(U/ml) | | TBIL/3m  (umol/L) | | FIB(g/L) | |
|  | XGBoost | 0.18 | | 0.16 | | 0.16 | | | 0.22 | | 0.15 | | 0.13 | |
|  | RF | 0.28 | | 0.20 | | 0.20 | | | 0.11 | | 0.15 | | 0.07 | |

**REFERENCES**

Biau, G., Scornet, E. (2016) . A random forest guided tour. ***Test-Spain.*** 25, 197-227.

Babajide, M. I., Saeed, F. (2016). Bioactive Molecule Prediction Using Extreme Gradient Boosting. ***Molecules.*** 21, 983.

Cortez, P., Embrechts, M. J. (2013). Using sensitivity analysis and visualization techniques to open black box data mining models. ***Inform Sciences.*** 225,1-17.

Devos, O., Ruckebusch, C., Durand, A., Duponchel, L., Huvenne, J. (2009). Support vector machines (SVM) in near infrared (NIR) spectroscopy: Focus on parameters optimization and model interpretation. ***Chemometr Intell Lab.*** 96, 27-33.

Stoltzfus, J. C. Logistic Regression: A Brief Primer. (2011). ***Acad Emerg Med.*** 18, 1099-1104.
